# The Content of Anthocyanins in Cowpea (*Vigna unguiculata* (L.) Walp.) Seeds and Contribution of the *MYB* Gene Cluster to Their Coloration Pattern

**DOI:** 10.3390/plants12203624

**Published:** 2023-10-20

**Authors:** Ekaterina А. Krylova, Aleksandra S. Mikhailova, Yulia N. Zinchenko, Irina N. Perchuk, Mayya P. Razgonova, Elena K. Khlestkina, Marina O. Burlyaeva

**Affiliations:** 1 N.I. Vavilov All-Russian Institute of Plant Genetic Resources, B. Morskaya 42-44, 190000 Saint-Petersburg, Russia; a.mikhailova@vir.nw.ru (A.S.M.); yu-zinch@yandex.ru (Y.N.Z.); i.perchuk@vir.nw.ru (I.N.P.); m.razgonova@vir.nw.ru (M.P.R.); director@vir.nw.ru (E.K.K.); 2 Advanced Engineering School, Institute of Biotechnology, Bioengineering and Food Systems, Far Eastern Federal University, 10 Ajax Settlement, Russky Island, 690922 Vladivostok, Russia

**Keywords:** seed coat, anthocyanins, *MYB* gene, in silico, confocal laser scanning microscopy, biochemistry

## Abstract

The intensively pigmented legumes belonging to *Phaseolus* and *Vigna* spp. are valued as an essential component of healthy nutrition due to their high content of flavonoids. In this context, we used the accessions of *Vigna unguiculata* with different colors of seed coats from the N.I. Vavilov All-Russian Institute of Plant Genetic Resources collection as the main object of this research. We applied confocal laser scanning microscopy, biochemical analysis, and wide in silico and molecular genetic analyses to study the main candidate genes for anthocyanin pigmentation within the *MYB* cluster on chromosome 5. We performed statistical data processing. The anthocyanin content ranged from 2.96 mg/100 g DW in reddish-brown-seeded cowpea accessions to 175.16 mg/100 g DW in black-seeded ones. Laser microscopy showed that the autofluorescence in cowpea seeds was mainly caused by phenolic compounds. The maximum fluorescence was observed in the seed coat, while its dark color, due to the highest level of red fluorescence, pointed to the presence of anthocyanins and anthocyanidins. Genes of the *MYB* cluster on chromosome 5 demonstrated a high homology and were segregated into a separate clade. However, amplification products were not obtained for all genes because of the truncation of some genes. Statistical analysis showed a clear correlation between the high content of anthocyanins in cowpea seeds and the presence of PCR products with primers Vigun05g0393-300-1.

## 1. Introduction

*Vigna unguiculata* (L.) Walp. (cowpea) is a warm-season grain legume. This crop plays a crucial role in the world’s agriculture, as well as in the human diet. Cowpea is a multipurpose crop, grown mostly for seeds or green vegetable pods. Cowpea is most widely used as a grain crop. Seeds are rich in protein, minerals, vitamins, folic acid, and many other nutrients, including lysine and tryptophan, which are absent in cereals [1,2]. Consumers prefer large-seeded light-colored cowpea varieties. However, it is known that legumes contain the largest amount of polyphenols in seeds with dark, intensive pigmentation of the seed coat, which mainly belongs to *Phaseolus* L. and *Vigna* Savi species. A high content of phenols usually correlates with the highest antioxidant activity [3,4,5]. In recent years, a large amount of information has been accumulated about the antioxidant, anticarcinogenic, antibacterial, and other types of activity of the phenolic compounds contained in the seeds of these species [6,7,8,9,10,11]. For humans, these substances are interesting as bioactive compounds with beneficial properties [12]. The main groups of phenolic compounds in *Vigna* are phenolic acids and flavonoids [2,6]. Flavonoids are one of the main classes of secondary metabolites in plants and fungi [13,14]. They play an important role in protecting plants from abiotic and biotic stresses [15,16,17,18,19]. Flavonoids include several subclasses of compounds (catechins, flavones, flavonols, flavan-4-ols, leucoanthocyanidins, anthocyanidins, etc.). Some of them are responsible for plant tissue coloration [20,21,22,23,24]. In particular, anthocyanins are the main coloring substances in plants, and this property allows them to dye plant tissues in various shades—from red to purple and blue [16,25,26,27]. Cultivars with colored seeds, pods, and sprouts have a specific dietary value [28,29,30,31,32]. Colored seeds in the legume family are rich in phenolic components that have an antioxidant effect due to their ability to trap free radicals and exhibit antimutagenic and anticarcinogenic activities [29,30,33,34]. The anticarcinogenic effect of phenolic compounds in *V. unguiculata* can manifest itself in the form of protecting DNA from damage and in suppressing the proliferation of cancer cells [6,35]. Some flavonoids can act as prooxidants, and perhaps in this way they are involved in the coordination of cellular functions.

The regulation of flavonoid biosynthesis, in particular anthocyanins, in *V. unguiculata* seeds has been studied by a number of researchers. The first studies into the inheritance of cowpea seed color were started at the beginning of the last century using classical genetic methods [36,37,38]. Spillman identified two factors, W and H, that determine the seed color [38]. Subsequently, Harland continued studying the inheritance of anthocyanin color in flowers, seeds, and cowpea pods [36,37]. He supposed that the combination of four factors (B, N, M, and R) determined the seed color. Later, the efforts to study the inheritance of cowpea seed color were continued. Candidates of regulatory and structural genes were identified [39,40,41,42,43].

The variety of flavonoids is formed as a result of phenylpropanoid and flavonoid biosynthesis pathways that control two groups of genes—structural and regulatory [44,45]. Structural genes encode biosynthesis enzymes, while regulatory genes encode transcription factors (TFs) that control the expression of structural ones through binding with the *cis*-acting DNA elements in the promoter region of the target sites [46,47,48]. Transcription control of flavonoid biosynthesis pathways is undertaken by the ternary MBW complex (R2R3-MYB, bHLH-Myc, and WD40 families) [49,50,51]. MYB proteins can be divided into three subfamilies according to the number of adjacent repeats in the MYB domain at the N-terminus: one (R0), two (R1), three (R2), or four (R3) [52]. Each repeat consists of approximately 50–53 amino acids and encodes three *α*-helices, with the second and third helices forming a helix–turn–helix (HTH) structure, which binds with the major groove of DNA [53,54,55,56]. In the genome of *Arabidopsis thaliana* (L.) Heynh., there are 125 *R2R3-MYB* genes that control many processes in plants, such as the regulation of many secondary metabolism stages, in particular flavonoid pigment biosynthesis [57,58]. For example, the early biosynthetic genes in *A. thaliana* are positively regulated by three R2R3-MYB proteins (MYB11, MYB12, and MYB111) [59]. The expression of the late biosynthetic genes is under control of the whole MBW complex that includes four R2R3-MYB TFs (MYB75/PAP1 [PRODUCTION OF ANTHOCYANIN PIGMENT1], MYB90/PAP2, MYB113, or MYB114), one WD-repeat protein TTG1 (TRANSPARENT TESTA GLABRA1), and three bHLH TFs (GL3, EGL3, and TT8) [60]. AtMYB123 (TRANSPARENT TESTA2 [TT2]), TT8, and TTG1 regulate proanthocyanidin accumulation in *A. thaliana* developing seed coats [61,62]. Some MYB proteins (AtMYB4, AtMYB7, AtMYBL2, AtCPC) play a negative role in regulating anthocyanin biosynthesis in Arabidopsis [63,64]. AtMYB4 and AtMYB7 repress the flavonoid biosynthesis by negatively regulating the expression of dihydroflavonol 4-reductase (DFR) and UDP-galactose flavonoid 3-O-galactosyltransferase genes [65]. AtMYBL2 negatively regulates the expression of DFR and TT8 to regulate anthocyanin biosynthesis [66]. Single-repeat R3 MYB transcription factors such as CPC are known to play roles in root hair differentiation and trichome initiation. Moreover, it competes with the R2R3-MYB transcription factor PAP1/2 and can influence anthocyanin pigment synthesis. The regulation of MYB activators and repressors maintains the balance in the accumulation of anthocyanin in different parts of plants. MYB transcription factors are considered to be key components that provide specificity for the downstream genes and play an important role in the process of tissue-specific anthocyanin accumulation [63].

Deeper knowledge about the patterns of flavonoid accumulation is necessary to solve the problem of expanding the diversity of plant products. The identification and characterization of genes that control flavonoid biosynthetic pathways are important for successful breeding of modern cultivars with increased dietary value. This will speed up the selection of varieties with improved seed quality for use in nutraceutical nutrition. At the moment, the role of the *MYB* gene cluster to cowpea seed coat coloration pattern has not been determined. The aim of the study was to determine the contribution of the *MYB* gene cluster to the cowpea seed coat color and to identify a correlation with the anthocyanin content of the seeds.

## 2. Results

### 2.1. Confocal Laser Scanning Microscopy

Laser microscopy exploits the ability of chemicals to fluoresce when excited by a laser and makes it possible to locate certain groups of chemical compounds in plant tissues. We applied this method to visualize the spatial arrangement of compounds in the seed coat on the basis of autofluorescence.

**Figure 1:**
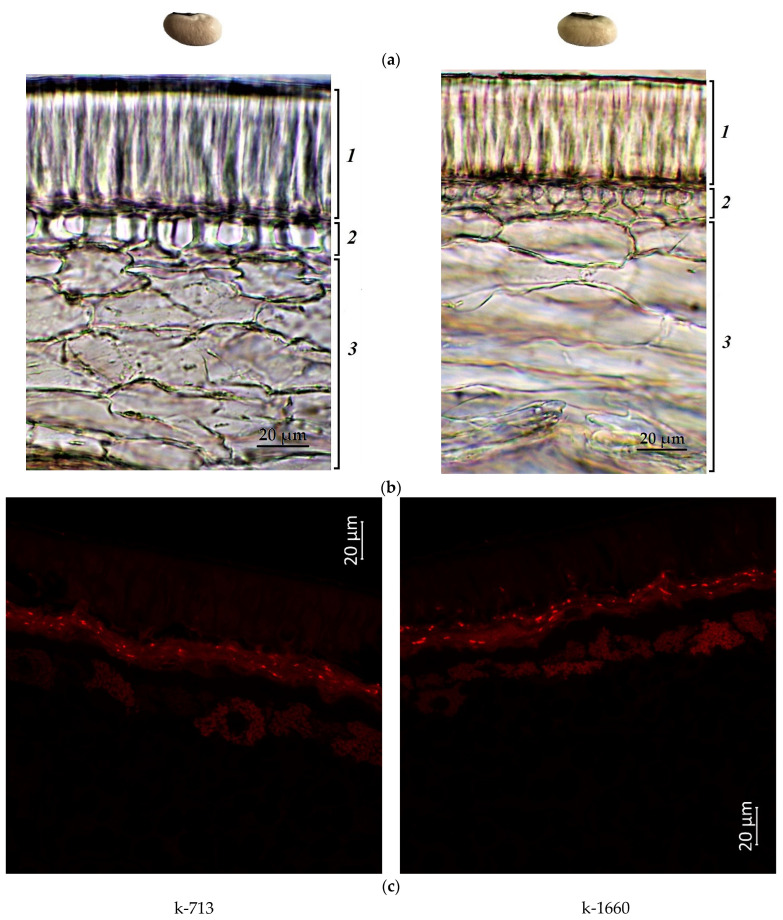
*V. unguiculata* accession: (**a**) seed; (**b**) seed coat structure, light microscopy (1—palisade layer, 2—hypoderma, 3—parenchyma); (**c**) transverse section of the seed, confocal microscopy, excitation at 488 nm with an emission of 620–700 nm.

**Figure 2:**
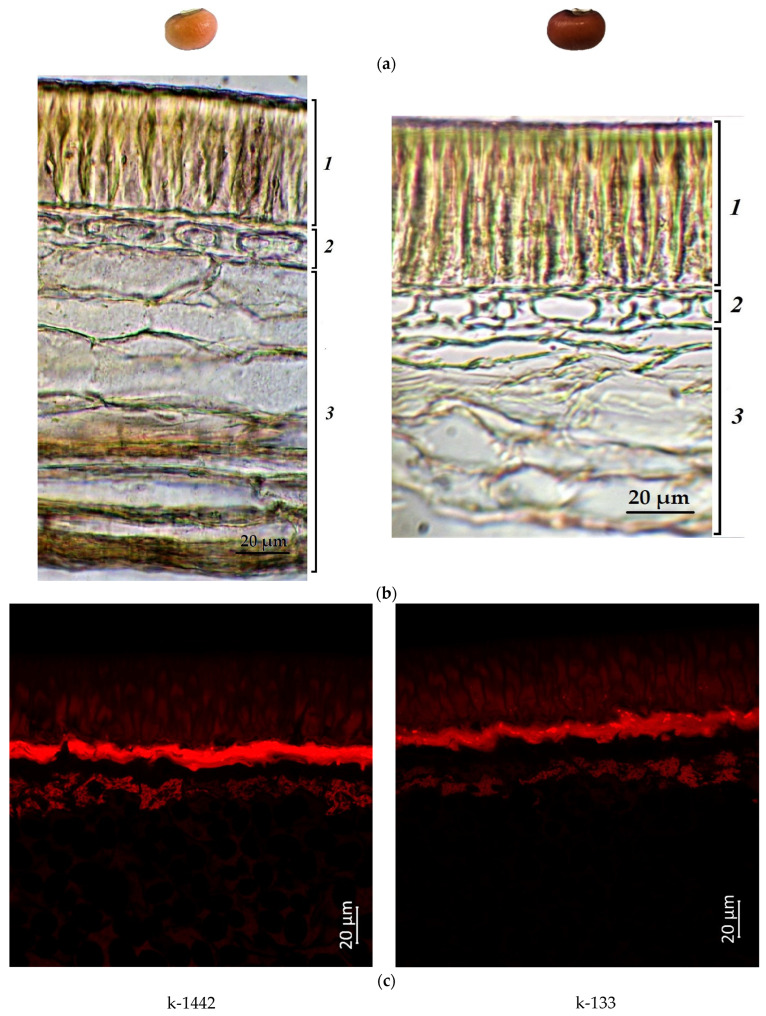
*V. unguiculata* accession: (**a**) seed; (**b**) seed coat structure, light microscopy (1—palisade layer, 2—hypoderma, 3—parenchyma); (**c**) transverse section of the seed, confocal microscopy, excitation at 488 nm with an emission of 620–700 nm.

**Figure 3:**
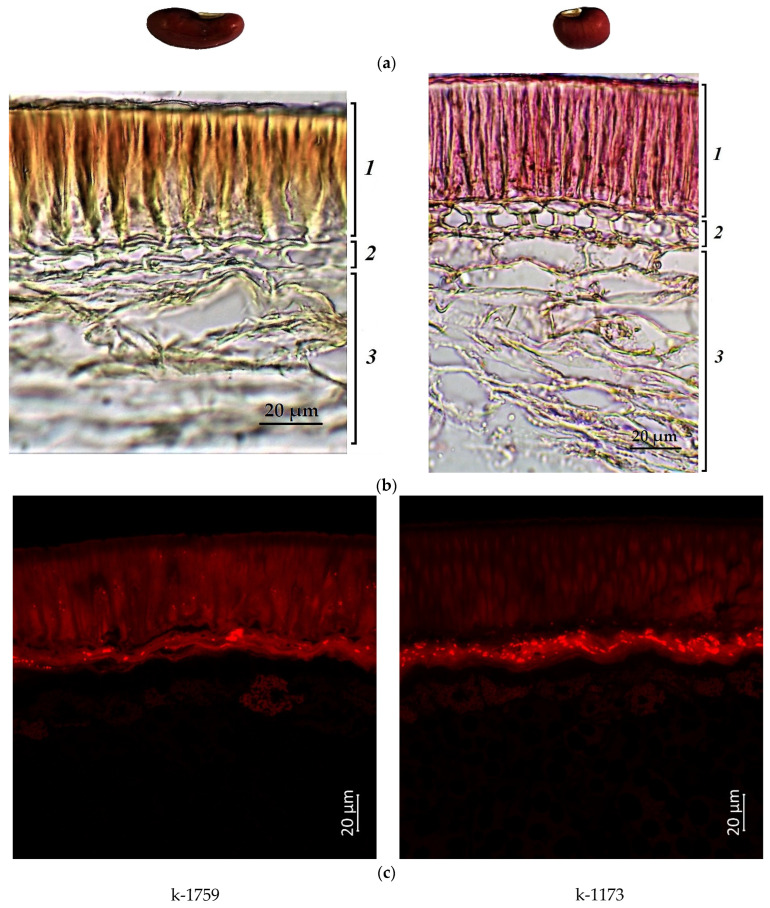
*V. unguiculata* accession: (**a**) seed; (**b**) seed coat structure, light microscopy (1—palisade layer, 2—hypoderma, 3—parenchyma); (**c**) transverse section of the seed, confocal microscopy, excitation at 488 nm with an emission of 620–700 nm.

**Figure 4:**
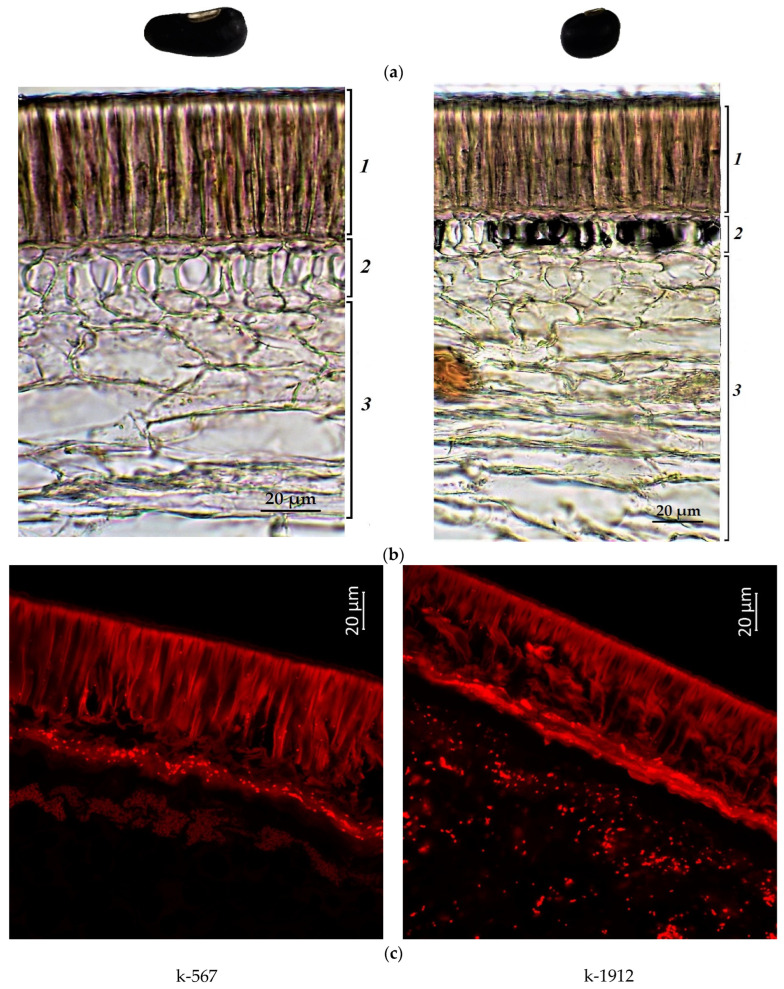
*V. unguiculata* accession: (**a**) seed; (**b**) seed coat structure, light microscopy (1—palisade layer, 2—hypoderma, 3—parenchyma); (**c**) transverse section of the seed, confocal microscopy, excitation at 488 nm with an emission of 620–700 nm.

Figure 1, Figure 2, Figure 3 and Figure 4 illustrate the transverse sections of cowpea seeds under a confocal laser microscope. The palisade layer was clearly visible in the seed coat, and there were several adjacent thin layers under it, which were difficult to differentiate from each other. Each cotyledon was covered with a single layer of epidermal cells. The parenchyma cells of the cowpea cotyledon contained small protein bodies in an unstructured protein matrix. The big rounded or elliptical non-fluorescent structures in the cotyledon cells were starch granules [67,68].

Moreover, the confocal laser microscopy showed the presence of red fluorescent substances in the cowpea seeds.

The main emitters of the red fluorescence in non-green plant tissues were anthocyanins and anthocyanidins [69,70,71]. In general, the present study revealed that the darker the seed color was, the more pronounced the red fluorescence signal was. The white-seeded accessions (k-713 and k-1660) showed a weak red fluorescence, visible mainly in the lower layers of the seed coat (hypoderma and parenchyma). A very weak signal was observed in the palisade layer of the seed coat and cotyledons (Figure 1).

Accession k-1442 (light-brown seeds) also had a weak red signal in the palisade layer (Figure 3). At the same time, a stronger red emission in this layer was demonstrated by accession k-133 (brown seeds) (Figure 2), which indicated the presence of anthocyanins and/or anthocyanidins, and/or other phenolic compounds.

The accessions (k-1173 and k-1759) with reddish-brown seeds showed bright red fluorescence in the lower layers of the seed coat. In addition, more pronounced fluorescence was observed in the palisade layer, especially in k-1759 (Figure 3).

The black seeds had the brightest red fluorescence among the studied accessions. It could be observed in all cells of the seed coat of both black-seeded k-567 and k-1912 (Figure 4). It is known that the black color of the seed coat in legumes is induced by a large amount of anthocyanins [72]. This study confirmed that the bright red fluorescence in black seeds was caused by those pigments.

The present microscopic analysis revealed that anthocyanins were not found in a noticeable amount in the cotyledon epidermis of all studied samples of any color. However, a red glow was observed in the cotyledons in the first layer of cells under the epidermis among all accessions. A strong red glow was detected in all cells of the cotyledons in the black-seeded k-1912, while anthocyanins were located as scattered inclusions.

### 2.2. Anthocyanin Content in Cowpea Seeds

Anthocyanin-free accessions had white seeds (k-713 and k-1660). The *OD*_510_*–OD*_657_ in these accessions was 0.032. The *OD*_510_*–OD*_657_ in the accessions with light-brown and brown seeds (k-133 and k-1442) was 0.032–0.039. These values did not differ significantly from the white-seeded control. Therefore, they were attributed to the accessions that had no anthocyanins.

The content of anthocyanins in seeds was calculated for the accessions with an *OD*_510_*–OD*_657_ that was greater than the control. The concentration of anthocyanins in the powder obtained from seeds ranged from 2.56 to 175.16 mg/100 g DW (Table 1).

In reddish-brown seeds (k-1759 and k-1173), the concentration of anthocyanins was 2.56–2.94 mg/100 g. The highest content of anthocyanins was in black seeds (k-567 and k-1912): 90.88–175.16 mg/100 g.

### 2.3. Identification and Phylogenetic Analysis of MYB-like Genes in Cowpea

In the framework of this study, a search for homologous sequences of the *AtMYB114* gene of *A. thaliana* was carried out in the cowpea genome. MYB proteins can be classified into three subfamilies according to the number of adjacent repeats in the MYB domain (one, two, or three). It is known that there are two unusual genes encoding proteins with two or more repeats: AtMYBCDC5 (GenBank: AT1G09770, ARABIDOPSIS THALIANA CELL DIVISION CYCLE 5) and AtMYB4R1 (GenBank: AT3G18100, MYB DOMAIN PROTEIN 4R1), which is a putative MYB protein containing four R1R2-like repeats. AtMYBCDC5 contains an MYB domain consisting of two repeats that are only distantly related to those of the R2R3-type MYB domain. The similarity of this domain to a typical R2R3-type MYB domain was 31%, whereas R2R3-type MYB domains generally have at least 40% similarity to the consensus [57]. AtMYBCDC5 may have a function in cell cycle regulation [73].

The *AtMYBCDC5* gene was used as the outgroup.

**Figure 5:**
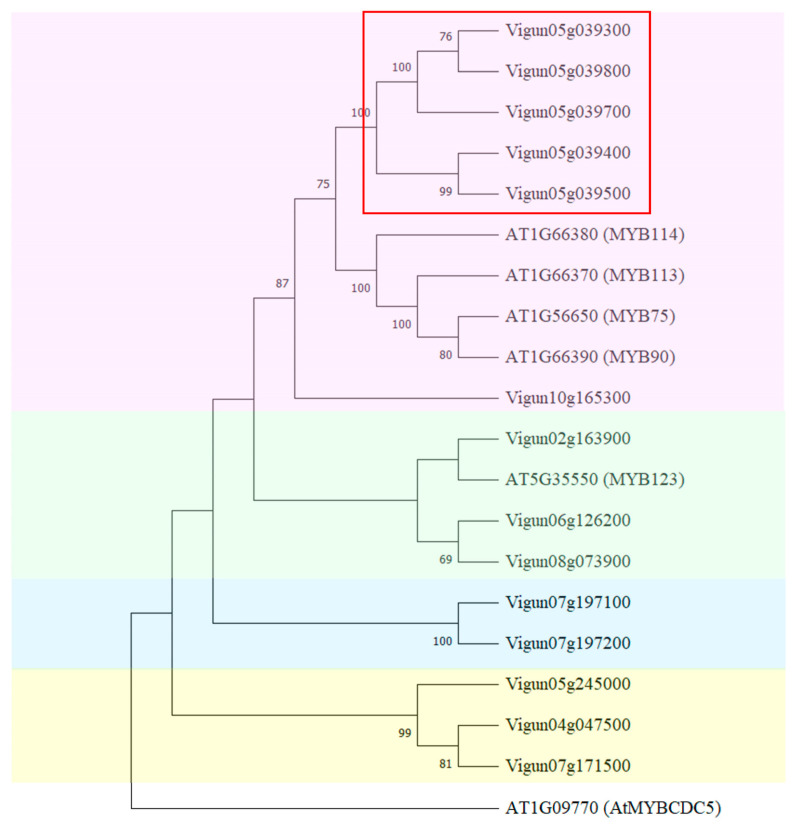
Analysis of phylogenetic similarity among *MYB*-like genes (CDS). The phylogenetic tree was reconstructed in MEGA X using the Neighbor-Joining method with 1000 bootstrap replicates. The genes in the MYB cluster on chromosome 5 from *V. unguiculata* are highlighted with a red rectangle. The *AtMYBCDC5* gene (GenBank: AT1G09770) was used as the outgroup.

In total, we identified fourteen sequences of *AtMYB114*-like genes in the genome of *V. unguiculata*. As shown in Figure 5, all identified sequences were divided into four clades.

The first clade included *AtMYB113*, *AtMYB114*, *AtMYB90*, *AtMYB75*, and sequences highly homologous to the *AtMYB114* gene of *A. thaliana* (Figure 5, pink). In this clade there was a cluster of *MYB* genes on chromosome 5: *Vigun05g039300*, *Vigun05g039400*, *Vigun05g039500*, *Vigun05g039700*, and *Vigun05g039800*.

*Vigun05g039500* was highly homologous to *Vigun05g039400*; these genes formed one group with high bootstrap replicates (Figure 5). According to the transcriptomic atlas of cowpea (accessed on 1 December 2021), a high level of expression of these two genes was observed in developing seeds [74]. The expression level of *Vigun05g039500* was greater than that of *Vigun05g039400*. The other three genes in the cluster on chromosome 5 had different expression patterns. A high expression level of *Vigun05g039300* was observed in flowers, developing pods, and the stem; while for the *Vigun05g039800* gene, its expression was detected in leaves and lower in the stem. For *Vigun05g039700*, no expression was found in any tissues. In this clade the *Vigun10g165300* gene had high expression levels in flowers and the stem, and a lower level of transcript accumulation in developing seeds.

The second clade (Figure 5, green) was formed by sequences highly homologous to *AtMYB123*, a regulator of proanthocyanidin biosynthesis. In the cowpea genome, such genes were located on chromosomes 2, 6, and 8. The expression of *Vigun02g163900* was detected in flowers and pods; meanwhile, a high level of expression of *Vigun06g126200* and *Vigun08g073900* was observed in developing seeds, pods, and other tissues.

The next two clades (Figure 5, blue and yellow) were formed by five sequences. The *Vigun07g197100* and *Vigu07g197200* genes were highly homologous to each other, and they were grouped in a distinct clade (blue) with high bootstrap replicates. These genes had different expression patterns; however, the expression of both genes was found in seeds.

The last clade included three genes located on chromosomes 4, 5, and 7. The expression of the *Vigun05g245000* gene was observed only in roots. Meanwhile, the expression of *Vigun04g047500* was noted in developing seeds, and transcript accumulation of *Vigun07g171500* genes was registered in many tissues, in particular, at a high level in seeds and pods.

The structural organization of the identified genes was determined. The exon–intron structure of most identified *AtMYB114*-like cowpea genes was the same. The majority of the identified genes consisted of three exons and two introns. The exceptions were *Vigun06g126200* and *Vigun08g073900* which consisted of two exons and one intron.

### 2.4. Annotation of the Functional Domains and Prediction of 3D MYB-Cluster Protein Structures

During pairwise comparison of amino acid sequences in *A. thaliana* (MYB113, MYB114, and MYB90) and MYB-like *Vigna* proteins, we established that in all cases their homology exceeded 50% (Table 2). It is important to note that the homology of Vigun05g039300 and Vigun05g039800, as well as that of Vigun05g039700 and Vigun05g039800, was higher than in other pairs and reached 96.1% and 94.8%, respectively.

**Figure 6:**
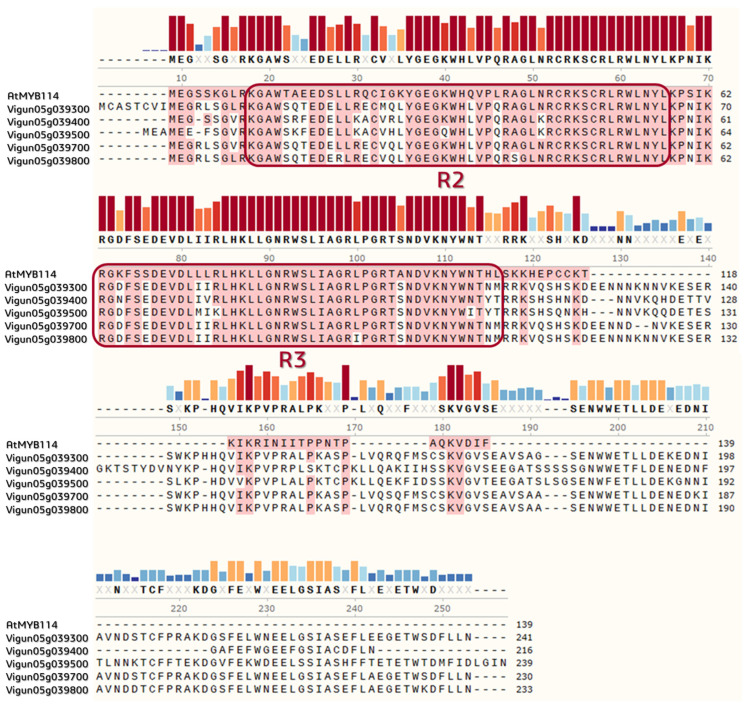
Multiple alignment of MYB-like DNA-binding proteins in the *V. unguiculata* genome with *A. thaliana* AtMYB114 (GenBank: NP_001321376), generated using ClustalW within the SnapGene Viewer 6.0.7 software. The colored bars show sequence conservation with red highlighting identical residues; motifs-containing R2/R3 MYBs are united at the frame.

Multiple alignment of the MYB-like DNA-binding proteins in the *V. unguiculata* genome with *A. thaliana* AtMYB114 showed that all considered genes were related to a family of MYB-like DNA-binding domains (InterPro: PF00249) and had R2/R3 conservative motifs with a length of 46 amino acids (Figure 6).

As previously established, all predicted proteins belonged to the MYB transcriptional factor family.

The 6KKS 3D structure of the R2R3-type MYB transcription factor (https://www.rcsb.org/structure/6KKS, accessed on 12 June 2022) was used as a model template to predict 3D proteins in *V. unguiculata*. Deposited in the SWISS-MODEL repository, the *A. thaliana* transcription factor WER (WEREWOLF) was related to the R2R3-MYB family and was encoded by the *AtMYB66* gene (R2R3-Myb family; GenBank: AT5G14750), specifically interacting with the major groove of the DNA [56].

**Figure 7:**
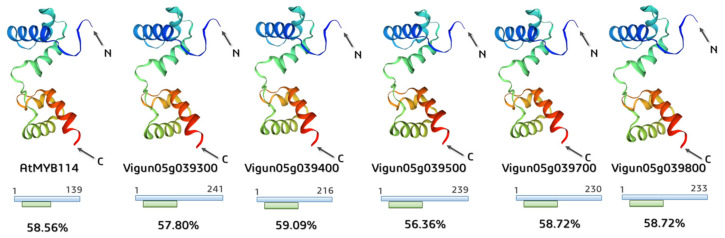
Predicted 3D structures of *A. thaliana* and *V. unguiculata MYB*-like proteins (6KKS template from PDB). The blue line under 3D structures shows the length of the investigated protein sequence, while the green line under 3D structures shows its coverage by the applied templates. The identity of amino acid sequences between the model AtMYB66 protein and AtMYB114, including *Vigna* proteins, is also presented.

The applied template covered the conservative R2R3-motif region among all structures; the identity between amino acid sequences of the model AtMYB66 and AtMYB114 and *Vigna* proteins reached approximately 57.7% on average (Figure 7).

### 2.5. Amplification and Resequencing of the MYB-Cluster Genes on Chromosome 5

Firstly, we performed amplification with primers for *Vigun04g203000* which encode actin to establish the suitability of the extracted total DNA. We accomplished amplification of genes in the *MYB* cluster on chromosome 5 and sequencing of *Vigun05g039500*. PCR was conducted for cowpea accessions with different seed coat colors (from white to black, see Section 4.1).

Amplification of the *Vigun05g039300* gene was successful only for two black-seeded accessions, k-567 and k-1912 (Table 3 and Table 4). It is important to note that the amplification was successful regardless of the primers used. For other accessions, the results of *Vigun05g039300* amplification were different. Amplification with only one primer pair (300-2) was successful in one of the white (k-713), light-brown (k-1442), brown (k-133), and reddish-brown (k-1173) accessions. These primers (300-2) flanked the coding sequence of the *Vigun05g039300* gene. For the remaining two accessions (white k-1660 and reddish-brown k-1759), amplification was detected only with the primers Del-st-F, upstream approximately 4456 bp of the *Vigun05g039300* gene, and 300-R, downstream of the *Vigun05g039300* gene. Thus, this gene was truncated in the genomes of these six accessions. Two black-seeded accessions were exceptions, with a non-truncated *Vigun05g039300* gene.

Successful amplification of the *Vigun05g039400* gene was attained only in one white-seeded (k-1660), one reddish-brown (k-1759), and two black-seeded (k-567 and k-1912) accessions (Table 3 and Table 4). The same amplification pattern was found for the *Vigun05g039500* gene. Successful amplification with all used primer pairs again was registered only for one reddish-brown (k-1759), one white-seeded (k-1660), and two black-seeded (k-567 and k-1912) accessions (Table 3 and Table 4). Thus, we procured amplification of this gene not only in black-seeded accessions. To search for allelic differences, we performed sequencing of the part of the *Vigun05g039500* gene that contained coding R2/R3 conservative motifs, in the accessions with successful amplification. We did not find any SNPs, insertions or deletions in the sequenced part of the *Vigun05g039500* gene (Figure S1). This fact indicates that this gene has a conservative sequence. Amplification of *Vigun05g039500* failed in one of the white (k-713), light-brown (k-1442), brown (k-133), and reddish-brown (k-1173) accessions.

Gene amplification was performed for *Vigun05g039700* as well. As with *Vigun05g039300*, the amplification of *Vigun05g039700* was always detected only in two black-seeded accessions, k-567 and k-1912 (Table 3 and Table 4). It is important to note that amplification was successful regardless of the primers used. However, for accession k-567, we detected deletion in 253 bp in the non-coding region (Figure S2). This deletion was flanked by a Del-end-F/Del-st-R primer pair, near the *Vigun05g039700* gene. For two accessions (white k-1660 and reddish-brown k-1759), amplification was successful only with the 700-1 primers that flanked the coding sequence of the *Vigun05g039700* gene. At the same time, amplification with the primers Del-end-F and Del-st-R, which was performed downstream of the *Vigun05g039700* gene, failed. For the remaining accessions, on the contrary, amplification of *Vigun05g039700* was detected only with the primers Del-end-F and Del-st-R, but not with 700-1. In this connection, we concluded that this gene was truncated in the genome of those six accessions. Two black-seeded accessions were exceptions: they had a non-truncated *Vigun05g039700* gene.

In accordance with the above, only black-seeded accessions had all genes in the *MYB* cluster on chromosome 5. Two non-black-seeded accessions (white k-1660 and reddish-brown k-1759) had only the *Vigun05g039300* gene truncated, while other genes in the *MYB* cluster were retained. The other four non-black-seeded accessions (white k-713, light-brown k-1442, brown k-133, and reddish-brown k-1173) had truncated *Vigun05g039300* and *Vigun05g039700* genes, and missed the *Vigun05g039400* and *Vigun05g039500* genes.

### 2.6. Statistical Analysis

**Figure 8:**
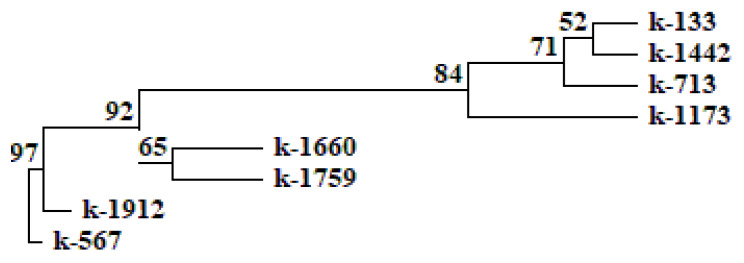
UPGMA (Unweighted Group Average or Unweighted Pair Group Method with Arithmetic Averaging) dendrogram constructed on the basis of a comparative analysis of anthocyanins, their distribution in the cotyledons and seed coat, and the studied genes of the MBW complex in *V. unguiculata* seeds.

**Figure 9:**
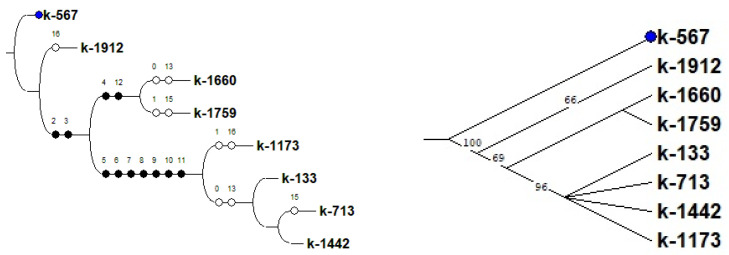
Consensus tree built using the criterion of maximum parsimony based on the results of a comparative analysis of anthocyanins, their distribution in the cotyledons and seed coat, and genes of the MBW complex (L = 24, Ci = 70, consistency index is the proportion of homoplasia in the total number of changes in traits; Ri = 81, retention index is the number of synapomorphies determined by the data). Black circles denote unifying features, white circles denote distinguishing features. 0–2—Different range of anthocyanin content; 3–12—amplification results for *Vigun05g039300*, *Vigun05g039400*, *Vigun05g039500*, and *Vigun05g039700*; 13, 15, 16—presence of anthocyanins in the palisade layer, first cell-layer under the epidermis in cotyledons, and all cotyledon cells, respectively.

The comparison of the data obtained as a result of the biochemical analysis and confocal laser scanning microscopy, along with the analysis of MBW-complex genes using statistical methods, revealed a clear relationship between the high content of anthocyanins in cowpea seeds and the successful amplification of the Vigun05g0393-300-1 product. A UPGMA dendrogram (Unweighted Group Average or Unweighted Pair Group Method with Arithmetic Averaging) was constructed on the basis of a comparative analysis of anthocyanins, their distribution in the cotyledons and seed coat, and the studied genes of the MBW complex in *V. unguiculata* seeds. On the UPGMA dendrogram and consensus tree (built using the criterion of maximum parsimony), black-seeded accessions k-567 and k-1912 were located most closely together (Figure 8 and Figure 9). These accessions were distinguished by the highest content of anthocyanins and the presence of all studied genes in the non-truncated form (*Vigun05g039300*, *Vigun05g039400*, *Vigun05g039500*, and *Vigun05g039700*). The only difference between these accessions was the absence of anthocyanin in the cotyledons of k-567 (results of confocal microscopy, Figure 4). In other accessions, there was no clear relationship between the results of amplification in the studied genes, the seed color, and the content of anthocyanins in the seeds. Thus, the accession with white seeds (anthocyanin-free k-1660) and the one with reddish-brown seeds (k-1759, with a low anthocyanin level) were combined into one clade. These accessions were grouped due to the presence of the *Vigun05g039400* and *Vigun05g039500* genes and the failed amplification of the *Vigun05g039300* gene (with primers 300-1 and 300-2) and the failed amplification of the fragment with Del-end-F/Del-st-R primers. The accessions with different seed colors (white k-713, brown k-133, light-brown k-1442, and reddish-brown k-1173) were grouped into another clade that had a fairly high level of bootstrap support (84%). The unifying factor for these accessions (anthocyanin-free and with a low anthocyanin level) was the failed amplification of the genes *Vigun05g039400*, *Vigun05g039500*, and *Vigun05g039700*, and the fragment with primers Del-st-F/300-1-R. These accessions had an unequal intensity of the glow of cells in different layers of the seed coat and cotyledons (Figure 1, Figure 2 and Figure 3).

A high consistency index (Ci = 70) on the consensus tree should be noted. This parameter points to high homoplasia, i.e., independent occurrence of mutations in different accessions, which leads to the same type of variability in traits. The high number of synapomorphies (Ri = 81) indicates the monophyletic nature of the studied group.

## 3. Discussion

Anthocyanins are one of the most thoroughly studied groups of plant pigments. Dark-colored fruits, such as blueberries and pomegranates, are the main sources of anthocyanins. These pigments are recognized as components of functional nutrition. With this in view, knowledge about genetic regulation is necessary today in the context of the initiated breeding programs aimed at increasing the content of anthocyanins in cultivated plants. One of the important plants is cowpea, a species widely cultivated in southern countries; it occupies the third place in the world in terms of the area of cultivation among leguminous crops. Investigations into the inheritance of cowpea seed color started at the beginning of the last century [36,37,38].

In this study, the anthocyanin content in cowpea seeds was strongly correlated with the seed coat color. Namely, the highest values were observed in black-seeded accessions (90.88–175.16 mg/100 g), and significantly lower values were recorded in reddish-brown-seeded ones (2.56–2.94 mg/100 g). If the data are compared with the results of a study of anthocyanins in black kidney bean or soybean seeds, the content of anthocyanins in black-seeded cowpea is much higher [4,72].

A fluorescence analysis made it possible to identify the features common to all the studied cowpea accessions. Various phenolic compounds were found to be responsible for autofluorescence, with the main contribution from hydroxycinnamic acids, flavonols, anthocyanins, and anthocyanidins [75]. Our study showed that the fluorescence of the cotyledon parenchyma cells was rather weak. Cowpea cotyledons contained more than 40% non-fluorescent starch [76]. In seeds of the majority of accessions (except for k-1912, which had high content of anthocyanins in all cells), only the outermost layer of the cotyledon cells under the epidermis showed a bright fluorescence. Other researchers previously observed a similar phenomenon in soybean seeds, noting that it required further study [77].

At the same time, the total autofluorescence was greatest in the seed coat of all accessions. Our results were consistent with numerous publications, indicating that the total concentration of phenolic compounds was always much higher in the seed coat than in the cotyledons of *Vigna* [78,79,80] and other legumes [81,82,83]. In accordance with our data, the largest number of anthocyanins accumulated in the cells of the hypodermis and palisade epidermis of the seed coat. The strongest red-light glow in the palisade layer was observed among the black-seeded accessions and the ones with the highest anthocyanin content.

The accumulation of phenolics mainly in the outer layers of the seed might be associated with their protective function during seed development, and against detrimental agents from the environment [84].

After a detailed characterization of the phenotype of the studied accessions, we studied the DNA polymorphism underlying the phenotypic differences described above.

Candidate genes, acting as regulatory genes of the MBW complex, were suggested using different methods [34,35]. The *VuCPC* (*Vigun11g115400*) and *VuMYB4* (*Vigun01g142900*) genes were identified in the cowpea genome. These genes were highly homologous to the *AtCPC* and *AtMYB4* genes of *A. thaliana*, and they were antagonists to anthocyanin biosynthesis [39,40,43]. The transcription factors of the MYB, bHLH-Myc, and WD40 families regulated anthocyanin biosynthesis through tissue-specific regulation of the expression of structural genes that encoded biosynthesis enzymes. The R2R3-MYB genes (*Vigun05g039300*, *Vigun05g039400*, *Vigun05g039500*, and *Vigun05g039700*) were identified on chromosome 5. A deletion starting from *Vigun05g039300* with the end in *Vigun05g039700* was detected in non-black accessions. Herniter et al. supposed that the size of this deletion may be different. *Vigun05g039500* was supposed to be the candidate gene for the black seed coat of cowpea [41].

In the present study, we identified genes that were highly homologous to *AtMYB113*, *AtMYB114*, *AtMYB90*, and *AtMYB75* (Figure 5). These genes were regulatory and were involved in the anthocyanin biosynthesis pathways. The *MYB* cluster on chromosome 5 consisted of the R2R3-Myb genes that were highly homologous to *AtMYB114*. Moreover, due to the alignment of multiple amino acids of the MYB-like proteins in *Vigna*, we revealed that strongly conservative residues were retained (Figure 6). For instance, the key tryptophan (W) residues, forming the hydrophobic cluster to stabilize the spatial helix–turn–helix structure of each repeat, were spotted at positions 13, 33, and 53 in the R2-motif [85,86]. This pointed to the preservation of the DNA-binding activity among the considered MYB proteins in *Vigna*.

In the present study, the amplification results of the MYB-cluster genes varied depending on cowpea accessions. In the accessions with black seeds, *Vigun05g039500* sequences were very conservative, so this gene was present in two studied black-seeded accessions. These results were consistent with the data obtained earlier [34]. PCR products with different primers to *Vigun05g039500* were not observed in some non-black accessions (one white-seeded anthocyanin-free control, accessions with light-brown and brown seeds lacking anthocyanins, and one reddish-brown-seeded accession with low anthocyanin content). The size of the deletion on chromosome 5 within the MYB cluster was different in non-black-seeded accessions. Some accessions had a truncated *Vigun05g039300*, while others had *Vigun05g039300* and *Vigun05g039700*.

It could be assumed that seeds with a small amount of anthocyanins did not need this gene in the non-truncated state. It was amplified in some accessions with all primer pairs. This fact may indicate that, although *Vigun05g039500* was complete, another TF-bHLH-type Myc was possibly defective in these accessions. Again, the sequence of *Vigun05g039500* was very conservative in these accessions, and the results of sequencing confirmed it. Therefore, the seed color of these accessions might be due to the transcription factor bHLH-Myc. In the accessions with the black seed coat, both genes were obviously functional. The *Vigun07g110700* gene was determined as a basic helix–loop–helix gene, and *Vigun09g139900* as WD40. *Vigun10g163900* encoded E3 ubiquitin ligase [42]. It was located near to the R2R3-MYB *Vigun10g165300* gene that had a high-level expression in the flowers and stem, and a lower level of transcript accumulation in developing seeds. The function of this gene in the regulation of seed coloration pattern cannot be excluded. Furthermore, it is necessary to establish the role of the other identified R2R3-MYB genes in the regulation of proanthocyanidin and anthocyanin biosynthesis pathways. The individual combinations of bHLH and R2R3 MYB proteins regulate the expression of different downstream genes. These complexes determine the specific pattern of pigment accumulation in different plant parts. In addition, the role of genes (*VuCPC* (*Vigun11g115400*) and *VuMYB4* (*Vigun01g142900*)) with negative roles in regulating anthocyanin biosynthesis should not be excluded.

The UPGMA dendrogram and the consensus tree revealed a clear relationship between the high content of anthocyanins and the presence of the *Vigun05g0393-300-1* amplification product only in the black-seeded cowpea. In non-black accessions (white-seeded anthocyanin-free control, light-brown and brown accessions lacking anthocyanins, and reddish-brown accessions with low anthocyanin content) this amplification product was absent. It is probable that light-brown and brown seed coat colors depend on the accumulation of compounds other than anthocyanins.

Understanding the DNA polymorphism underlying the variability in seed coat cowpea color requires not only a deeper study of the MYB-cluster genes on chromosome 5, but also necessitates an analysis of the genes in the bHLH-Myc family. Such data are of particular importance for the initiated breeding programs aimed at increasing the content of anthocyanins in cultivated plants.

## 4. Materials and Methods

### 4.1. Materials

We used the seeds of *V. unguiculata* from the collection of the N.I. Vavilov All-Russian Institute of Plant Genetic Resources (VIR) (Table 5).

**Figure 10:**
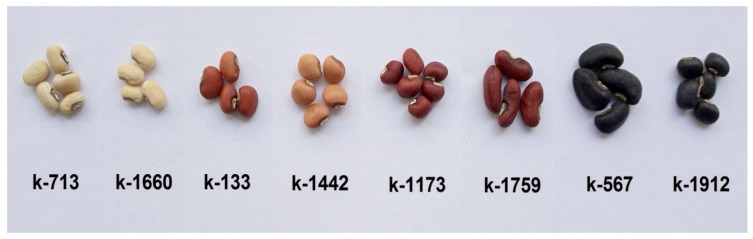
Seed appearance of the studied cowpea accessions.

The accessions with different seed coat colors were selected for the analysis: white (k-713 and k-1660), brown (k-133), light brown (k-1442), reddish brown (k-1173 and k-1759), and black (k-567 and k-1912) (Figure 10). Samples from the same year and reproductive site (2017 year, Astrakhan Experiment Station of the N.I. Vavilov All-Russian Institute of Plant Genetic Resources, Saint-Petersburg, Russia) were included in research. For analysis, seeds of all shades that are characteristic for this accession were selected. The seed set for each accession was representative and reflected all diversity of seed coat color intensity.

### 4.2. Confocal Laser Scanning Microscopy

Untreated cowpea seeds were used for microscopic examination. Five randomly selected seeds of each accession were used for research. The transverse dissection of seeds was performed with an MS-2 sledge microtome (Tochmedpribor, Kharkiv, Ukraine). Autofluorescence of anthocyanins was observed using a confocal laser scanning microscope (LSM 800, Carl Zeiss Microscopy GmbH AG, Berlin, Germany). According to the published data, anthocyanins should be registered in the red region of the spectrum [58,59,60]. The samples were excited at 488 nm with the emission in 620–700 nm. The objective lens Plan-Apochromat 63×/1.40 Oil DIC M27 with 63× magnification and the ZEN 2.1 software (Carl Zeiss Microscopy GmbH, Berlin, Germany) were used for image acquisition and processing.

### 4.3. Assessment of Anthocyanin Content in Seeds

Cowpea seeds with various coat colors were used for the analysis. An average seed sample weighing 25–30 g (100–150 seeds) was milled on the Lab.mill-1 QC-114 device. The content of anthocyanins was measured using the spectrophotometric method in at least three analytical replicates (three each accession pre-ground seeds samples 0.5–1.0 g were taken for analysis). Thus, each accession was represented by three samples.

The total content of anthocyanins was assessed using Muravieva’s method with some modifications [87]. Briefly, 0.5–1 g of the crushed sample was ground in a porcelain mortar with 15 mL of 1% HCl aqueous solution, moved to a glass vessel with a lid, and kept for 2 h at room temperature. The resulting extract was filtered through a Buchner funnel with a paper filter; the filter was washed with a small amount of 1% HCl solution. The volume of the obtained extract was measured. Part of the extract was centrifuged for 15 min at 12,000–15,000 rpm on the MPW-310 centrifuge. The optical density of the supernatant was measured on an Ultroshec II LKB spectrophotometer in a cuvette with a layer thickness of 10 mm at a wavelength of 510 nm to measure anthocyanins, and at 657 nm to correct for the content of green pigments. A 1% HCl aqueous solution was used as a comparison solution. The content of total anthocyanins in raw materials (*X*, mg/100 g dry weight [DW]) in terms of cyanidin-3,5-diglycoside was calculated (for samples with *OD*_510_*–OD*_657_ significantly different from the anthocyanin-free control) according to the following formula:

$$X=\frac{OD\;*\;V\;*\;100\;*\;1000}{453\;*\;m\;*\;(100-W)}$$

where *OD = OD*_510_*–OD*_657_; *OD*_510_ is the solution absorption at a wavelength of 510 nm; *OD*_657_ is the solution absorption at a wavelength of 657 nm; *V* is the volume of the obtained extract (mL); 453 is the specific absorption index of cyanidin-3.5-diglycoside at a wavelength of 510 nm in a 1% aqueous solution of hydrochloric acid; *m* is the weight of the sample (g); and *W* is the weight loss during drying of raw seeds (%).

The anthocyanins content for every accession was calculated as the average value obtained for these three samples.

### 4.4. Identification of Genes and Phylogenetic Analysis, Prediction of 3D Protein Structures

Homologous sequences of *AtMYB114* (GenBank: *AT1G66380*) were searched for in the genome of *V. unguiculata* using the BLASTN algorithm in the databases Phytozome v13 (https://phytozome.jgi.doe.gov/pz/portal.html#, accessed on 10 June 2022) and LIS (Legume Information System; https://legumeinfo.org, accessed on 1 June 2022) [88,89]. Multiple alignment of nucleotide and amino acid sequences was made using the MULTALIN v5.4.1 (http://multalin.toulouse.inra.fr/multalin/, accessed on 12 June 2022) and SnapGene Viewer 6.0.7 software (available at https://www.snapgene.com/, accessed on 12 June 2022) [90]. A cluster analysis was performed with the help of the MEGA-X software Version 11.0.10 and the Neighbor-Joining algorithm with 1000 bootstrap replicates [91,92,93,94]. The resulting image showed bootstrap accounts more than ≥50%. The *AtMYBCDC5* gene (GenBank: AT1G09770) was chosen as the outgroup. The annotation of the functional domains was carried out relying on the collection of protein families in the Pfam database (http://pfam.xfam.org/, accessed on 12 June 2022) [95]. Modelling the tertiary structure of the predicted amino acid sequences was accomplished using the SWISS-MODEL (https://swissmodel.expasy.org/interactive, accessed on 12 June 2022) homology-modelling server based on the 6KKS template from the PDB [96]. The percent protein sequence identity was established with the LALIGN tool (https://fasta.bioch.virginia.edu/fasta_www2/fasta_www.cgi?rm=lalign&pgm=lal, accessed on 14 June 2022). The default settings were applied for all software.

### 4.5. DNA Extraction

Total genomic DNA was extracted from 5-day-old fresh germinated seedlings using the DNA-Extran-3 kit (Syntol, Moscow, Russia) according to the manufacturer’s instructions. The quality of the isolated DNA was evaluated in 1% agarose gel prepared on the basis of the TAE buffer (40 mM Tris-HCl, pH 8.0; 20 mM sodium acetate; 1 mM EDTA). Ethidium bromide was used as an intercalating dye. The Sky-High 250 b–10 kb DNA ladder (BioLabMix, Moscow, Russia) was applied as a molecular-weight size marker. The resulting products of amplification were visualized with the use of the Bio-Rad ChemiDoc MP Gel Documentation System (Bio-Rad Laboratories, Moscow, Russia). The extracted total DNA concentrations were determined with the help of a NanoDrop™ 2000/2000c One Microvolume UV-Vis Spectrophotometer (Thermo Fisher Scientific Inc., Waltham, MA, USA).

### 4.6. Primer Design and PCR Amplification, Sanger Sequencing

The primer design for amplification gene sequences was made using the IDT PrimerQuest Tool (http://eu.idtdna.com/PrimerQuest/Home, accessed on 15 June 2022). The list of the primers used is presented in Table 6. Amplification was made in 20 μL PCRs. Reaction mixtures contained 50–100 ng of genomic template DNA, 0.5 mM of each of the primers, 0.25 mM of each dNTP, 1× reaction buffer (67 mM TrisHCl, pH 8.8; 2 mM MgCl_2_; 18 mM (NH_4_)_2_SO_4_; 0.01% Tween 20), and 1–2.5 U Taq polymerase. DNA templates were amplified with initial denaturation at 95 °C for 2 min, and 35 cycles were run at 95 °C for 30 s, 50–62 °C for 30 s, and 72 °C for 1–2 min, followed by a final elongation at 72 °C for 5 min. PCR products were separated on agarose gels with ethidium bromide. The amplified fragments were purified from an agarose gel using the diaGene kit (Dia-m, Moscow, Russia) according to the manufacturer’s instructions. DNA sequencing was performed using the BigDye™ Terminator v3.1 Cycle Sequencing Kit (Applied Biosystems™, Waltham, MA, USA).

The sequence data obtained in our research have been submitted to NCBI/GenBank data libraries with accession numbers OR597571 (k-1660, gene *Vigun05g039500*), OR597572 (k-1759, gene *Vigun05g039500*), OR597572 (k-1912, gene *Vigun05g039500*), OR597573 (k-567, gene *Vigun05g039500*), OR614450 (k-1912, intergenic region between *Vigun05g039700* and *Vigun05g039800* genes), and OR614451 (k-567, intergenic region between *Vigun05g039700* and *Vigun05g039800* genes, deletion region).

### 4.7. Statistical Data Processing

Statistical analysis included a compilation of binary matrices for each successful amplification of the studied genes in *V. unguiculata* seeds, in which the “presence” (1) or “absence” (0) of the gene was recorded for each of the studied samples. Features associated with the presence of phenolic compounds in different layers of the seed coat (according to the results of confocal laser scanning microscopy) were coded in the same way. To confirm the presence of anthocyanins, we applied the Mann–Whitney test (U-test) and Student’s test (*t*-test) for comparing *OD*_510_*–OD*_657_ values of the extracts from the colored seeds with *OD*_510_*–OD*_657_ values of the extracts from the anthocyanin-free white-seeded control. For samples with *OD*_510_*–OD*_657_ significantly higher than in the control, we calculated the anthocyanin content. The content of anthocyanins in seeds was ranked and coded in 3 ranges: in the first range, anthocyanin content was less than 2.5 mg/100 g DW; in the second range, anthocyanins content was greater than 2.5 mg/100 g DW but less than 90.8 mg/100 g DW; in the third range, anthocyanins content was greater than 90.8 mg/100 g DW. Based on the total matrix, a dendrogram was built, demonstrating the relationship between the studied samples. The method of unweighted pair-group cluster analysis with arithmetic averaging (UPGMA) using the TREECON program was applied to construct the dendrogram. In addition, a consensus tree was predicted using the Winclada-Nona program with the maximum parsimony criterion. The tree was based on the results of a comparative analysis of successful amplification of the studied genes in *V. unguiculata* seeds and distribution of phenolic compounds in different seed tissue layers.

## 5. Conclusions

The highest content of anthocyanins was found in black-seeded accessions (up to 175.16 mg/100 g DW). Most of these compounds were localized in the palisade epidermis of the seed coat. Anthocyanins were not detected in white-seeded accessions. Light-brown and brown seeds were lacking anthocyanins. A low content of anthocyanins was found in reddish-brown seeds.

Only black-seeded accessions had all genes in the *MYB* cluster on chromosome 5, but the *Vigun05g039500* gene was required not only for black seed coat color. Successful amplification of this gene was also recorded in one reddish-brown (k-1759) and one white (k-1660) accession. The pattern of the other PCR products was different. Two non-black-seeded accessions (white k-1660, and reddish-brown k-1759) had a truncated *Vigun05g039300* gene, while other genes in the *MYB* cluster were retained. The other four non-black-seeded accessions (white k-713, light-brown k-1442, brown k-133, and reddish-brown k-1173) had truncated *Vigun05g039300* and *Vigun05g039700* genes, and missed the *Vigun05g039400* and *Vigun05g039500* genes.

Thus, to understand the main pattern of seed coat coloration, it is necessary to further study the genes of the MYB cluster on chromosome 5, and, in addition, to analyze the genes of the bHLH-Myc family. Furthermore, it is necessary to study the role of the *Vigun11g115400* and *Vigun01g142900* genes that play a negative role in regulating anthocyanin biosynthesis. The study of a larger number of samples will make it possible to understand the role of the regulatory genes of the MBW complex in the control of the biosynthesis of phenolic compounds. This will speed up the selection of varieties with improved seed quality for use in nutraceutical nutrition.

## Figures and Tables

**Table 1:** The content of anthocyanins in cowpea seeds.

Accession	Seed Color	*OD*_510_*–OD*_657_ *	Criterion, *p* **		Anthocyanins (mg/100 g DW)
			Student’s *t*-Test	Mann–Whitney U Test	
k-713	white	0.032 ± 0.003	–	–	–
k-1660	white	0.032 ± 0.001	–	–	–
k-1442	light brown	0.032 ± 0.003	0.26	0.23	–
k-133	brown	0.039 ± 0.007	0.37	0.52	–
k-1759	reddish brown	0.065 ± 0.006	0.07 × 10^−4^	0.01	2.56 ± 0.05
k-1173	reddish brown	0.076 ± 0.005	0.01 × 10^−5^	0.05 × 10^−1^	2.94 ± 0.12
k-567	black	2.295 ± 0.201	0.01 × 10^−10^	0.05 × 10^−1^	90.88 ± 7.87
k-1912	black	4.788 ± 0.063	0.02 × 10^−14^	0.03	175.16 ± 4.61

* *OD*_510_ is the solution absorption at a wavelength of 510 nm, and *OD*_657_ is the solution absorption at a wavelength of 657 nm. ** Marked effects are significant at *p* < 0.05.

**Table 2:** Protein sequence identity of *A. thaliana* (MYB113, MYB114, and MYB90) and MYB-like *Vigna* proteins established with the LALIGN tool.

	AtMYB113	AtMYB114	AtMYB90	Vigun05g039300	Vigun05g039400	Vigun05g039500	Vigun05g039700	Vigun05g039800
AtMYB113	-	80.7%	70.8%	50.5%	57.6%	59.9%	62.7%	61.5%
AtMYB114	80.7%	-	87.9%	79.3%	78.4%	74.5%	71.2%	76.6%
AtMYB90	70.8%	87.9%	-	64.2%	62.1%	61.2%	64.4%	62.8%
Vigun05g039300	50.5%	**79.3%**	64.2%	-	57.6%	59.9%	62.7%	61.5%
Vigun05g039400	57.6%	**78.4%**	62.1%	65.2%	-	71.9%	66.8%	64.8%
Vigun05g039500	59.9%	**74.5%**	61.2%	69.2%	71.9%	-	69.0%	67.5%
Vigun05g039700	62.7%	**71.2%**	64.4%	**95.3%**	66.8%	69.0%	-	94.8%
Vigun05g039800	61.5%	**76.6%**	62.8%	**96.1%**	64.8%	67.5%	**94.8%**	-

Bold indicates the highest homology with AtMYB114 and MYB-like *Vigna* proteins.

**Table 3:** Amplification results for *Vigun05g039300*, *Vigun05g039400*, *Vigun05g039500*, and *Vigun05g039700* with different primer pairs.

VIR Catalogue Number	Color of Seed Coat	Presence or Absence of PCR Product with Primer Pairs									
		393-300-1	393-300-2	Del-st-F/300-1-R	394-400-1	395-500-1	395-500-2	395-500-3	395-500-4	397-700-1	Del-end-F/ Del-st-R
К-713	white	0	1	0	0	0	0	0	0	0	1
К-1660	white	0	0	1	1	1	1	1	1	1	0
К-1442	light brown	0	1	0	0	0	0	0	0	0	1
К-133	brown	0	1	0	0	0	0	0	0	0	1
К-1173	reddish-brown	0	1	0	0	0	0	0	0	0	1
К-1759	reddish-brown	0	0	1	1	1	1	1	1	1	0
К-567	black	1	1	1	1	1	1	1	1	1	1
К-1912	black	1	1	1	1	1	1	1	1	1	1

(1)—presence of PCR product, (0)—absence of PCR product.

**Table 4:** Amplification results for *Vigun05g039300*, *Vigun05g039400*, *Vigun05g039500*, and *Vigun05g039700*.

VIR Catalogue Number	Seed Coat Color	Gene			
		*Vigun05g039300*	*Vigun05g039400*	*Vigun05g039500*	*Vigun05g039700*
k-713	White				
k-1660	White				
k-1442	Light brown				
k-133	Brown				
k-1173	Reddish brown				
k-1759	Reddish brown				
k-567	Black				
k-1912	Black				

The blue cells indicate successful amplification; the pink cells—a truncated gene; colorless (empty)—failed amplification.

**Table 5:** The studied cowpea accessions.

No	VIR Catalogue Number	Type of Accessions	Country of Origin	Color of Seed Coat
1	k-133	Landrace	Russia	brown
2	k-567	Landrace	India	black
3	k-713	Landrace	Trinidad and Tobago	white
4	k-1173	Landrace	Somalia	reddish brown
5	k-1442	Landrace	Nigeria	light brown
6	k-1660	Landrace	France	white
7	k-1759	Landrace	Kyrgyzstan	reddish brown
8	k-1912	Landrace	Bolivia	black

**Table 6:** Gene-specific primers used in the present study.

Primer	Gene	Sequence (5′-3′)	Reference
300-1-F	*Vigun05g039300*	GGTGGTTTTGACGGTAAGCA	[41]
300-1-R		TGTCCAACTCCACACCTTGA	
300-2-F *		ATGTGTGCAAGCACTTGTGTG	
300-2-R *		TAGAGACACCAACTGCGAATAC	
400-1-F	*Vigun05g039400*	AGCATGATGAGACGACCGTA	[41]
400-1-R		CCAAACTCTTCGCCCCAAAA	
500-1-F	*Vigun05g039500*	TCCCTGATTGCAGGAAGACT	[41]
500-1-R		CCAATTCTCCGACCCTGATA	
500-2-F *		GGGTTGAAGAGATGCAGGAA	
500-2-R *		ACCTGCATATTCATTCACAAACA	
500-3-F *		ATCTCATCTAGCGGTGCTTATAC	
500-3-R *		ATCTACAACTCTTCCTGCATCTC	
500-4-F *		TTATCAGGGTCGGAGAATTGG	
500-4-R *		GTAATCTGGAGGAGGCAGAAT	
700-1-F	*Vigun05g039700*	ACAGGGTTATGCATTCTGAGC	[41]
700-1-R		GGGATGAGAGAGATGGCGAA	
Del-st-F		AGGGAAAGATGAGTGCAGGC	
Del-st-R		AGGTCGCCCAACTTCACATT	
Del-end-F		AGCCTCTGAGAATTGGTGGG	
Del-end-R	*Vigun05g039700*	GGGATGAGAGAGATGGCGAA	
Act-F	*Vigun04g203000*	TCAGGTGTCCAGAGGTGTTGTA	[97]
Act-R		ATGGTTGTGCCTCCTGAAAGTA	

* Primer design was performed in this study.

## Data Availability

The data presented in the current study are available in the article.

## Supplementary Materials

The following supporting information can be downloaded at: https://www.mdpi.com/article/10.3390/plants12203624/s1, Figure S1: Alignment of the deduced nucleotide acid sequences of *Vigun05g039500* gene in k-1759, k-1660, k-1912, and k-567 accessions generated using the MULTALIN v5.4.1; Figure S2: Alignment of intergenic region between *Vigun05g039700* and *Vigun05g039800* genes in k-1912 and k-567 accessions generated using the MULTALIN v5.4.1.
