# Supplementary material for: The Content of Anthocyanins in Cowpea (Vigna unguiculata (L.) Walp.) Seeds and Contribution of the MYB Gene Cluster to Their Coloration Pattern
Source: Plants (Basel). 2023 Oct 20;12(20):3624. doi: 10.3390/plants12203624 (PMC10609810; doi:10.3390/plants12203624)
Supplement: Supplementary file 1 [file plants-12-03624-s001.zip › Figure S1.pdf]

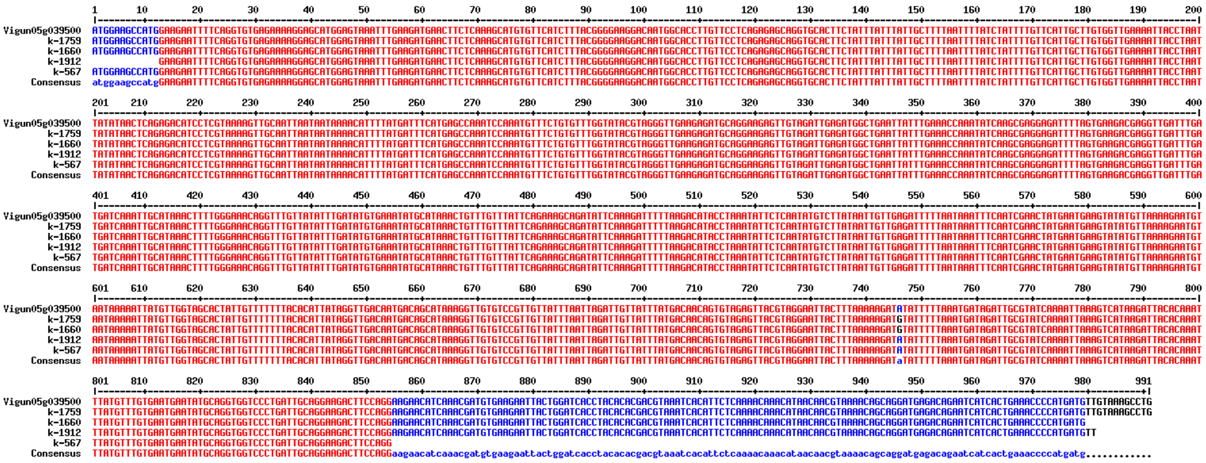

Figure S1. Alignment of the deduced nucleotide acid sequences of *Vigun05g039500* gene in k-1759, k-1660, k-1912 and k-567 accessions generated using the MULTALIN v5.4.1. Sequence of *Vigun05g039500* was from the database Phytozome v13.
