# Supplementary material for: The Content of Anthocyanins in Cowpea (Vigna unguiculata (L.) Walp.) Seeds and Contribution of the MYB Gene Cluster to Their Coloration Pattern
Source: Plants (Basel). 2023 Oct 20;12(20):3624. doi: 10.3390/plants12203624 (PMC10609810; doi:10.3390/plants12203624)
Supplement: Supplementary file 1 [file plants-12-03624-s001.zip › Figure S2.pdf]

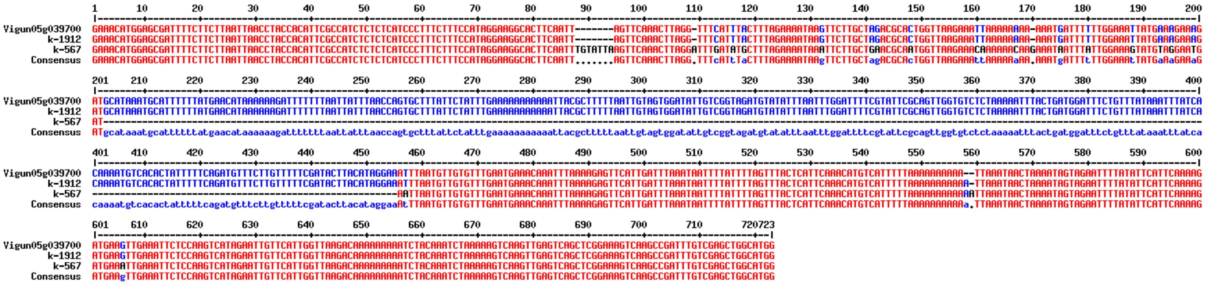

Figure S2. Alignment of intergenic region between *Vigun05g039700* and *Vigun05g039800* genes in k-1912 and k-567 accessions generated using the MULTALIN v5.4.1. Deletion in 253 bp was identified for accession k-567.
